# Growth and anemia among children with tuberculosis infection at different sites in Southwest China

**DOI:** 10.3389/fped.2023.1188704

**Published:** 2023-06-15

**Authors:** Zhongmin Gao, Quanbo Liu, Qin Deng, Lin Kong, Yongfang Liu

**Affiliations:** ^1^ Department of Nutrition, National Clinical Research Center for Child Health and Disorders, Ministry of Education Key Laboratory of Child Development and Disorders, Chongqing Key Laboratory of Child Health and Nutrition, Children’s Hospital of Chongqing Medical University, Chongqing, China; ^2^ Department of Infectious Diseases, Children's Hospital of Chongqing Medical University, Chongqing, China

**Keywords:** nutritional status, biochemical indexes, tuberculosis, children, China

## Abstract

**Objectives:**

To explore the effects of tuberculosis (TB) infection at different sites on anthropometric indicators, malnutrition and anemia incidence in children in Southwest China.

**Methods:**

From January 2012 to December 2021, a total of 368 children aged 1 month to 16 years were enrolled. According to the sites of TB infection, they were divided into three groups: tuberculous meningitis (T group), tuberculous meningitis complicated withpulmonary tuberculosis (TP group), and tuberculous meningitis complicated with pulmonary tuberculosis and abdominal tuberculosis (TPA group). Data on weight, height, nutritional risk, blood biochemical indicators and basic descriptions were collected within 48 h after admission.

**Results:**

The body mass index-for-age *z* score (BAZ), height-for-age *z* score (HAZ), and concentrations of hemoglobin (Hb) and albumin (ALB) decreased in the following order: T group, TP group, and TPA group. The prevalence of malnutrition was the highest in the TPA group (69.5%, 82/118) and 10-to 16-year-old group (72.4%, 63/87). Children aged 0.5–2 years exhibited the highest anemia prevalence of 70.6% (48/68) among the four age groups.The TPA group had the highest incidence of anemia (70.5%, 67/95) compared to T group and TP group.Compared with the treatment group, the abandonment group had a lower BAZ, HAZ and levels of HB and ALB, a higher rate of severe malnutrition, and higher nutritional risk scores. Children who had a low BAZ [odds ratio (OR) = 1.98], nutritional risk (OR = 0.56) and anemia (OR = 1.02) were less likely to obtain treatment with their guardians' support.

**Conclusions:**

Children with tuberculous meningitis were at risk for growth disorders and anemia, especially when complicated with pulmonary tuberculosis and abdominal tuberculosis. The prevalence of anemia and malnutrition was the highest among patients aged 1 month to 2 years and 10–16 years, respectively. Nutritional status was one of the causes of abandoning treatment.

## 1. Introduction

Tuberculosis (TB) is a chronic infectious disease that causes death worldwide. Childhood TB is considered a global pediatric emergency prevention disease (1). Approximately 9.9 million people suffered from TB and 1.3 million died from TB worldwide in 2020. Children under 15 years of age account for 11% of the total TB population but 15% of TB deaths (2). Although there are differences between countries, the burden of malnutrition and TB among children is the highest in Africa and Asia (3). China has the second largest number of TB cases in the world, with an estimated 0.84 million new TB cases in 2020, mainly distributed in poor areas in Southwest China (2). TBM is the most common and serious disease among children with extrapulmonary TB, followed by abdominal tuberculosis (ATB) (4, 5).

Malnutrition is common among children with TB. According to reports in the last 5 years, the incidence rates of malnutrition and severe malnutrition [weight-for-age *z* score (WAZ) < −3] were 49.1%−75.2% and 10.7–20.8%, respectively (6–9), in childhood TB. TB is closely related to nutrition (10). Malnutrition is increasingly recognized as a key risk factor for childhood TB (11). The World Health Organization (WHO) emphasized the association between malnutrition and TB in its roadmap for the diagnosis and treatment of childhood and adolescent TB (12). TB and malnutrition are mutually reinforcing and increase the prevalence and mortality of each other (13). Malnutrition affects the prognosis of TB and influences the growth and development of children. TB easily invades the intestine when ATB occurs, which directly affects the digestion and absorption of nutrients. Nutritional problems can be caused and exacerbated by inadequate dietary intake and intestinal absorption disorders in children with TB.

Nevertheless, the levels of growth and anemia among children with tuberculous meningitis (TBM) with or without pulmonary tuberculosis (PTB) and ATB have not been reported in Southwest China. Therefore, we conducted a cross-sectional survey in a large specialty children's hospital to understand the nutritional status of the patients mentioned above in Southwest China.

## 2. Materials and methods

### 2.1. Patients, inclusion criteria and grouping

This was a cross-sectional survey that was carried out between January 2012 and December 2021 in the Children's Hospital of Chongqing Medical University, which is a national Grade A children's hospital and approved as the national Children's Regional Medical Center. This hospital has the largest diagnosis and treatment center for infectious diseases and undertakes the diagnosis and treatment of infectious diseases in children in Southwest China, including Yunnan, Guizhou, Sichuan, Chongqing and Tibet. This study was approved by the Ethics Committee of the Children's Hospital of Chongqing Medical University in Chongqing, China.

The inclusion criteria were as follows: (a) children aged 1 month to 16 years; (b) children hospitalized for the first time; (c) children whose guardians signed a written informed consent form; Children with genetic metabolic diseases, congenital malformations of important organs, malignant tumors or other diseases with a known influence on physical growth were excluded from this study.

TBM was defined as follows: “Definite TBM” if AFB seen on culture from Cerebrospinal fluid (CSF) microscopy or mycobacterium tuberculosis (MTB) detected by culture from CSF; “Probable TBM” if the total score of ≥10 when neuroimaging was not available and a total score of ≥12 when neuroimaging was available (14). Children who fulfilled either of the two definitions of “Definite TBM” or “Probable TBM” as given above were included in the T group.

The diagnose of PTB included two types: “smear-positive PTB” if at least two sputum smear examinations positive for AFB or one sputum smear examination positive for AFB plus abnormal chest radiograph consistent with active PTB;“smear-negative PTB” if the clinical symptoms with ineffective broad-spectrum antibiotics treatment, effective anti-TB treatment, radiological abnormalities consistent with active PTB, and close contact with TB patients (15). Children who suffered from both TBM and PTB were included in the TP group.

Children diagnosed with ATB were divided into two types. “Confirmed case of ATB”—diagnosis based on the bacteriological identification of MTB through acid-fast stain and/or culture and/or polymerase chain reaction (PCR)—based assays or the presence of caseating granulomas on histology. “Clinically diagnosed ATB”—diagnosis based on exclusion of other diseases, with suggestive features on imaging(abdominal ultrasonogram and/or computed tomography), histology and biochemistry, and with effective response to anti-TB treatment (16). Children who suffered from TBM complicated with PTB and ATB were included in the TPA group.

According to the reasons for discharge (abandonment or recovery from illness), they were divided into treatment group and abandonment group.

### 2.2. Body measurements, blood biochemical indicators and nutritional risk screening

Weight was measured with undergarments by an electronic scale to the nearest 0.01 kg. Height was measured by a nonstretch measuring tape or height rod and marked to the nearest 0.1 cm. BMI was calculated according to the weight and height data, and the formula was as follows: BMI = weight (kg)/height (m)^2^. Anthropometric data were assessed as height-for-age *z* scores (HAZs) and BMI-for-age *z* scores (BAZs) using Anthro 2005, based on the WHO growth standards (http://www.who.int/childgrowth/software/en/). Cutoff points of <−1 standard deviation (SD) were used to define a low BAZ (malnutrition); −2 ≤ BAZ < −1 was used to define mild malnutrition; −3 ≤ BAZ < −2 was moderate malnutrition; and BAZ < −3 was severe malnutrition (17). BAZ < −2 indicated wasting.

Blood biochemical indicators were collected within 48 h after admission, including hemoglobin (Hb), total serum protein (TP), serum albumin (ALB), blood urea nitrogen (BUN), creatinine (Cr) and serum alkaline phosphatase (ALP). Hb levels were determined by the sodium dodecyl sulfate hemoglobin (SDS-Hb) determination method. TP,ALB, BUN and Cr concentrations were measured by biuret method,bromocresol green process,enzymatic method,and colorimetric method respectively. We used the kinetic rate approach that measured ALP activity by utilizing 2-amino-2-methyl-1-propanol (AMP) bufer.Anemia was defined based on the WHO guidelines (18) as follows: (1) children aged 6–59 months: Hb < 110 g/L; (2) children aged 5–11 years: Hb < 115 g/L; and (3) children aged 12–16 years: Hb < 120 g/L.

We used the Screening Tool for Risk on Nutritional status and Growth (STRONG) (19) to screen children for nutritional risk within 48 h after admission. This nutritional risk screening questionnaire consisted of 4 items: (1) nutritional intake and losses (1 point), including excessive diarrhea (≥5 times/day) and/or vomiting (>3 times per day) and reduced food intake during the last few days before admission; (2) subjective clinical assessment (1 point), including diminished subcutaneous fat and/or muscle mass and/or hollow face; (3) high-risk disease (2 points), including diseases with a risk of malnutrition or expected major surgery; and (4) weight loss or poor weight gain (1 point). According to the nutritional risk score, there were three levels of nutritional risk: low risk (0 points), moderate risk (1–3 points), and high risk (4–5 points).

### 2.3. Statistical analysis

Data were analyzed by SAS 9.4. The significance level was set at 5%. The Kolmogorov–Smirnov test was used to investigate whether the concentrations of Hb, TP, ALB, Cr, BUN, ALP, and the anthropometric indicators were normally distributed prior to analysis. Data are presented as the means, SDs and medians (interquartile ranges, IQRs). The means of 2 groups of continuous normally distributed variables were compared by independent sample t tests, while Tukey‒Kramer tests were used to compare the differences among the 3 groups of data in pairs. The Wilcoxon signed-rank test was used for nonnormally distributed data. Differences in prevalence were tested with a chi-square test. In the logistic regression analysis, continued treatment was the dependent variable (0 = no, 1 = yes). The final correction factors included in the logistic regression model were age, BAZ, anemia, degree of nutritional risk and diagnosis. The independent variables of each grade were assigned as follows: age (1 = <2 years old, 2 = 2–5 years old, 3 = 5–10 years old, 4 = 10–16 years old); BAZ (1 = BAZ < −3, 2 = −3 ≤ BAZ < −2, 3 = −2 ≤ BAZ < −1, 4 = BAZ ≥ −1); anemia (0 = yes, 1 = no); degree of nutritional risk (0 = low risk, 1 = moderate risk, 2 = high risk); and diagnosis (1 = TBM, 2 = TBM + PTB, 3 = TBM + PTB + ATB).

## 3. Results

### 3.1. Basic characteristics of participants

In total, 368 children met the eligibility criteria for this survey. The children were from different regions in Southwest China, with 322 (87.5%) living in rural areas and 46 (12.5%) living in urban areas; 208 (56.5%) were boys, while 160 (43.5%) were girls. There were 99 (26.9%) cases in the T group, 151 (41%) in the TP group, and 118 (32.1%) in the TPA group. As Table 1 shows, there was no significant difference among the three groups in terms of age. The average age was 3.83 ± 1.14 years, with 113 (30.7%) between 1 month–2 years, 73 (19.8%) between 2–5 years, 95 (25.8%) between 5–10 years, and 87 (23.6%) between 10–16 years (Table 2). All of the children had nutritional risk (nutritional risk score ≥ 1 point), 125 (34%) had high nutritional risk (4–5 points), and 243 (66%) had moderate nutritional risk (1–3 points) (data not shown).The education level of guardians of children with tuberculosis in this study was generally poor [Junior middle school or below: 83% (306/368)]. The guardian reported that the food intake of the child was only 30%–50% of the usual amount.

**Table 1:** BAZ, HAZ and biochemical indicators among patients in different groups.

Parameter	T group* (*n* = 99)	TP group^†^ (*n* = 151)	TPA group^#^ (*n* = 118)	P*^,†^	P*^,#^	*P* ^†,#^
Age (year)	5.75 (3.08–9.33)	4.08 (0.91–9.50)	4.79 (0.80–11.42)	0.23	0.68	0.42
BAZ	−1.13 ± 1.36	−1.14 ± 1.48	−1.47 ± 1.31	0.63	0.04	0.01
HAZ	−0.03 ± 0.77	−0.25 ± 0.87	−0.52 ± 0.98	0.05	<0.001	0.02
Hb (g/L)	112.98 ± 15.10	109.55 ± 15.33	101.73 ± 18.61	0.11	<0.001	0.0001
TP (g/L)	65.09 ± 6.48	66.84 ± 7.82	65.02 ± 10.09	0.10	0.95	0.07
ALB (g/L)	41.63 ± 4.97	40.43 ± 5.60	36.40 ± 7.76	0.13	<0.001	<0.001
Cr (g/L)	26.47 ± 9.93	28.49 ± 11.87	29.47 ± 10.77	0.16	0.05	0.47
BUN (g/L)	3.14 ± 1.30	3.43 ± 1.33	3.23 ± 1.35	0.09	0.62	0.22
ALP (g/L)	134.87 ± 55.54	142.31 ± 61.07	143.54 ± 72.12	0.36	0.32	0.87

BAZ, body mass index-for-age *z* score; HAZ, height-for-age; *z* score; Hb, hemoglobin; TP, total protein; ALB, albumin; Cr, creatinine; BUN, blood urea nitrogen; ALP, salkaline phosphatase.

^*,†^ : Comparison of the T group and TP group.

^*,#^ : Comparison of the T group and TPA group.

^†,#^ : Comparison of the TP group and TPA group.

**Table 2:** HAZ and biochemical indicators among children of different ages.

	1 month ≤ age < 2 years			2 years ≤ age < 5 years			5 years ≤ age < 10 years			10 years ≤ age < 16 years		
Parameters	T group (*n* = 16)	TP group (*n* = 53)	TPA group (*n* = 44)	T group (*n* = 26)	TP group (*n* = 30)	TPA group (*n* = 17)	T group (*n* = 37)	TP group (*n* = 35)	TPA group (*n* = 23)	T group (*n* = 20)	TP group (*n* = 33)	TPA group (*n* = 34)
HAZ	−0.40 ± 0.58 *	−0.58 ± 1.03 *	−1.04 ± 1.22^†^	−0.11 ± 0.78	0.09 ± 0.74	−0.26 ± 0.87	0.21 ± 0.77	0.15 ± 0.62	−0.09 ± 0.66	−0.06 ± 0.77*	−0.14 ± 0.38*	−0.97 ± 0.47^†^
TP (g/L)	62.7 ± 6.47	65.54 ± 7.28	64.47 ± 9.17	63.02 ± 6.36	67.56 ± 7.43	64 ± 7.6	65.87 ± 4.98	66.54 ± 7.87	63.11 ± 10.67	68.23 ± 7.81	68.6 ± 8.83	67.54 ± 11.71
ALB (g/L)	39.26 ± 5.46	39.46 ± 5.5	36.63 ± 7.28	40.06 ± 5.15	40.91 ± 5.47	38.26 ± 8.18	43 ± 3.88	41.06 ± 4.67	35.48 ± 8.86	43.06 ± 5.14*	40.89 ± 6.74*	35.78 ± 7.54^†^
Cr (g/L)	17.64 ± 6.41	19.75 ± 4.57	22.59 ± 7.5	22.57 ± 5.43	24.24 ± 5.86	25.65 ± 5.07	29.07 ± 8.53	30.45 ± 7.48	31.59 ± 8.86	33.79 ± 11.96	44.32 ± 11.42	38.87 ± 10.6
BUN (g/L)	2.31 ± 1.32	3.05 ± 1.24	3.04 ± 1.44	2.94 ± 1.26	3.26 ± 1.35	3.21 ± 1.28	3.51 ± 1.29	3.75 ± 1.43	3.36 ± 1.26	3.39 ± 1.18	3.85 ± 1.17	3.4 ± 1.34
ALP (g/L)	161.75 ± 65.91	169.61 ± 68.73	191.03 ± 86.38	120.05 ± 34.72	131.15 ± 41.75	133.87 ± 48.21	127.71 ± 35.75	129.94 ± 55.73	127.8 ± 42.62	145.88 ± 85.66	121.74 ± 54.54	97.58 ± 31.19

HAZ, height-for-age *z* score; TP, total protein; ALB, albumin; Cr, creatinine; BUN, blood urea nitrogen; ALP, salkaline phosphatase.

* ^†^Values within a row with different superscript characters are significantly different among the corresponding groups, with *P *< 0.05.

### 3.2. *z* score distributions of physical parameters and blood biochemical indicators

**Figure 1:**
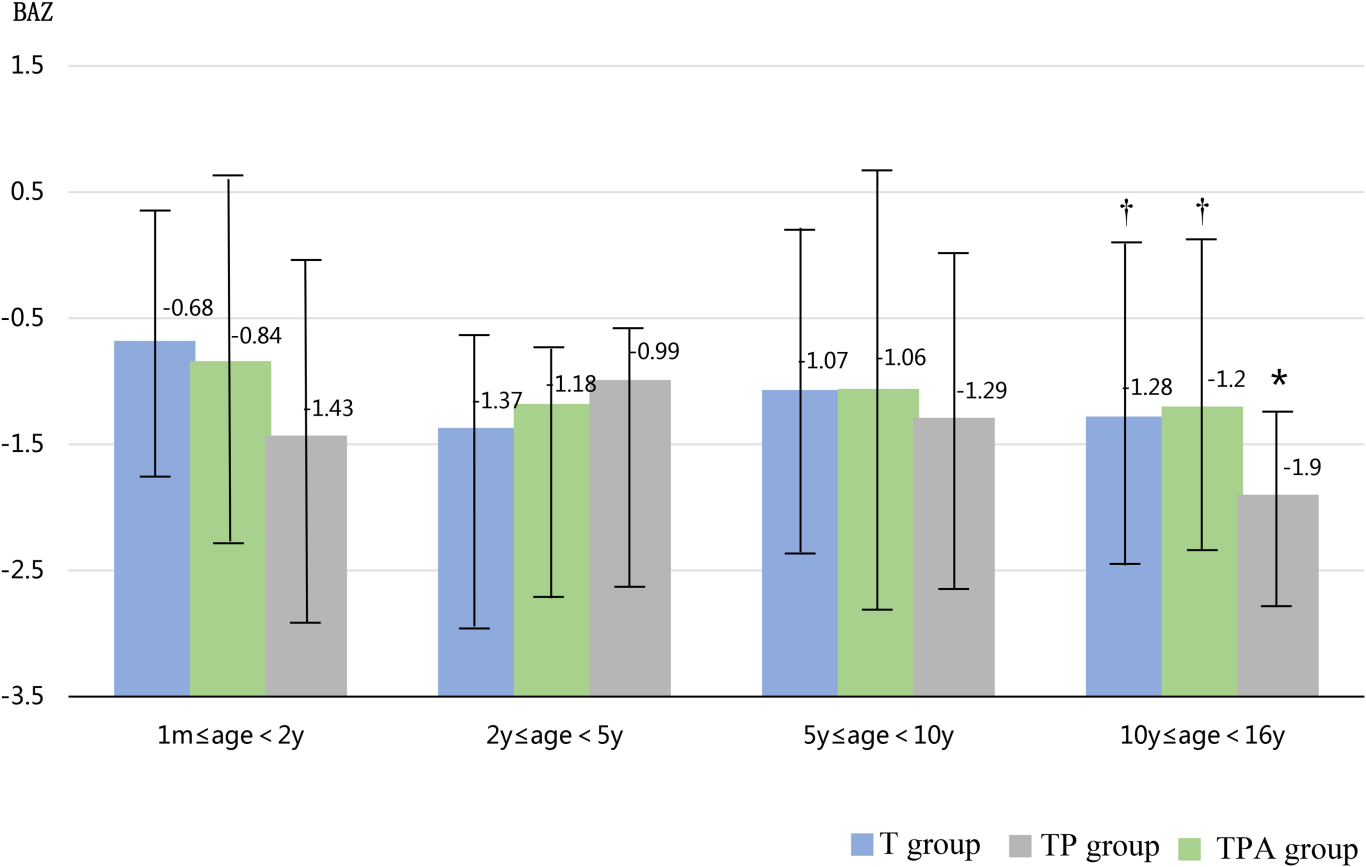
Comparison of BAZ levels in three groups at different ages. BAZ,body mass index-for-age z score. *Significant difference from other groups (p < 0.05).

**Figure 2:**
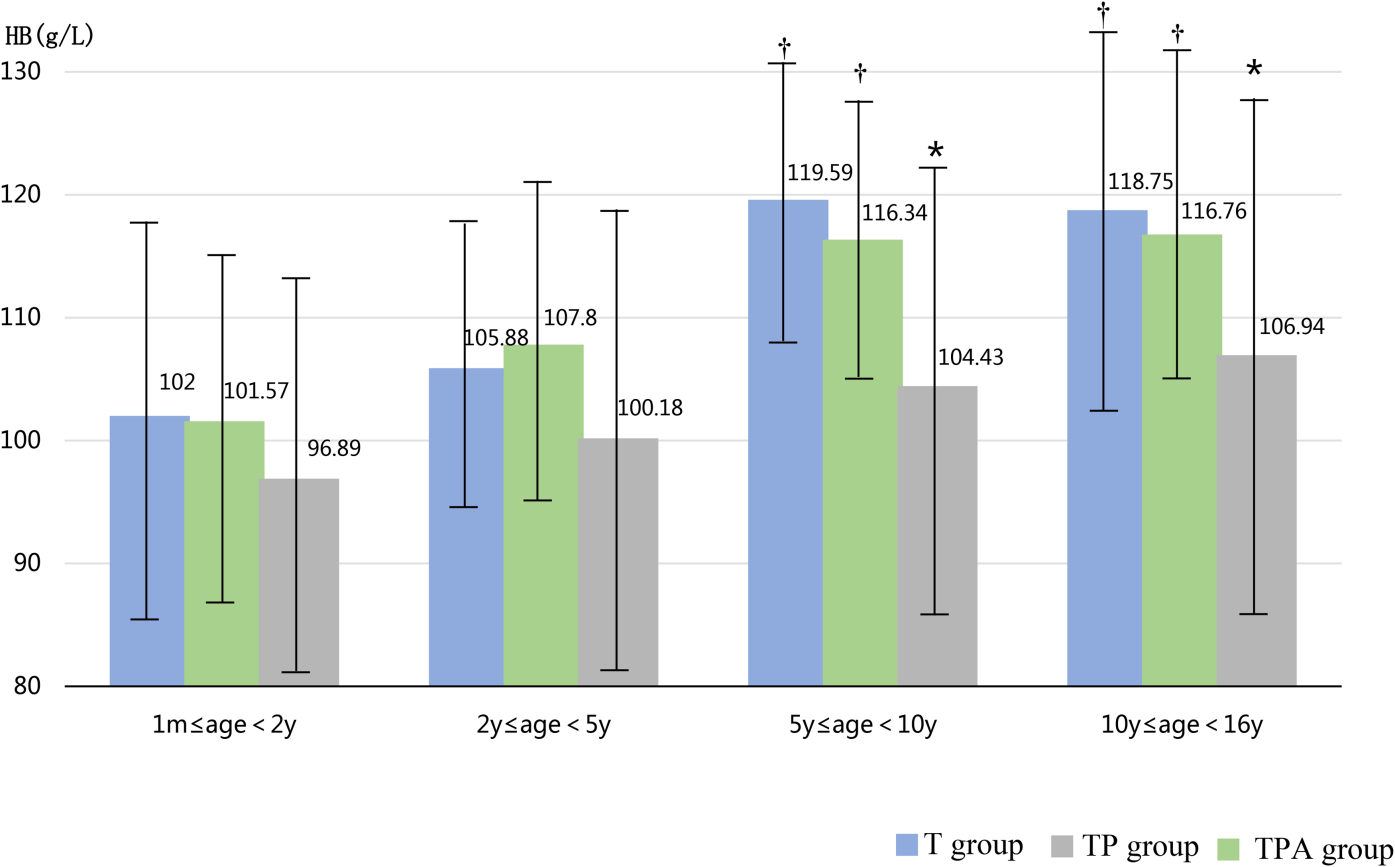
Comparison of HB levels in three groups at different ages. Hb,hemoglobin. *Significant difference from other groups (p < 0.05).

As shown in Table 1, the BAZ, HAZ, and concentrations of Hb and ALB decreased in the following order: T group, TP group, and TPA group. These were lower in the TPA group than in the other two groups, especially among children aged 10–16 years (Table 2, Figures 1, 2). Nevertheless, there were no significant differences in BAZs, HAZs or concentrations of Hb or ALB between the T group and TP group (*P *< 0.05). However, the ages of the children among the three groups were not significantly different. Furthermore, the concentrations of TP, Cr, BUN and ALP also showed no significant differences among the 3 groups.

### 3.3. Malnutrition and anemia

As shown in Table 3, the prevalence of malnutrition in the TPA group (82/118, 69.5%) was significantly higher than that in the T group and TP group (*P < *0.05). Similarly, the rate of malnutrition among children in the 10–16 age group (63/87, 72.4%) was higher than that in the other three age groups (*P < *0.05). However, there was no significant difference in the incidence rate of different degrees of malnutrition among the three groups and four age groups (*P *> 0.05).

**Table 3:** Malnutrition among the patients.

	Group			Age				*P* *	*P* ^#^
	T group (*n* = 99)	TP group (*n* = 151)	TPA group (*n* = 118)	1 month ≤ age < 2 years (*n* = 113)	2 years ≤ age < 5 years (*n* = 73)	5 years ≤ age < 10 years (*n* = 95)	10 years ≤ age < 16 years (*n* = 87)		
Degree of malnutrition									
Mild, *n* (%)	30 (30.3)	39 (25.8)	42 (35.6)	33 (29.2)	19 (26)	28 (29.5)	31 (35.6)	0.15	0.17
Moderate, *n* (%)	16 (16.2)	27 (17.9)	24 (20.3)	14 (12.4)	10 (13.7)	17 (17.9)	26 (29.9)		
Severe, *n* (%)	8 (8.1)	14 (9.3)	16 (13.6)	13 (11.5)	11 (15.1)	9 (9.5)	6 (6.9)		
Malnutrition, *n* (%)	54 (54.5)*	80 (53)*	82 (69.5)^†^	60 (53.1)*	40 (54.8)*	54 (56.8)*	63 (72.4)^†^	0.001	0.03

* ^†^Values within a row with different superscript characters are significantly different among the corresponding groups, with *P *< 0.05.

*P: Comparison of the T group, TP group, and TPA group.

^#^ *P*: Comparison of the four age groups. Malnutrition, BAZ < −1; mild malnutrition, −2 ≤ BAZ < −1; moderate malnutrition, −3 ≤ BAZ < −2; severe malnutrition, BAZ < −3.

**Figure 3:**
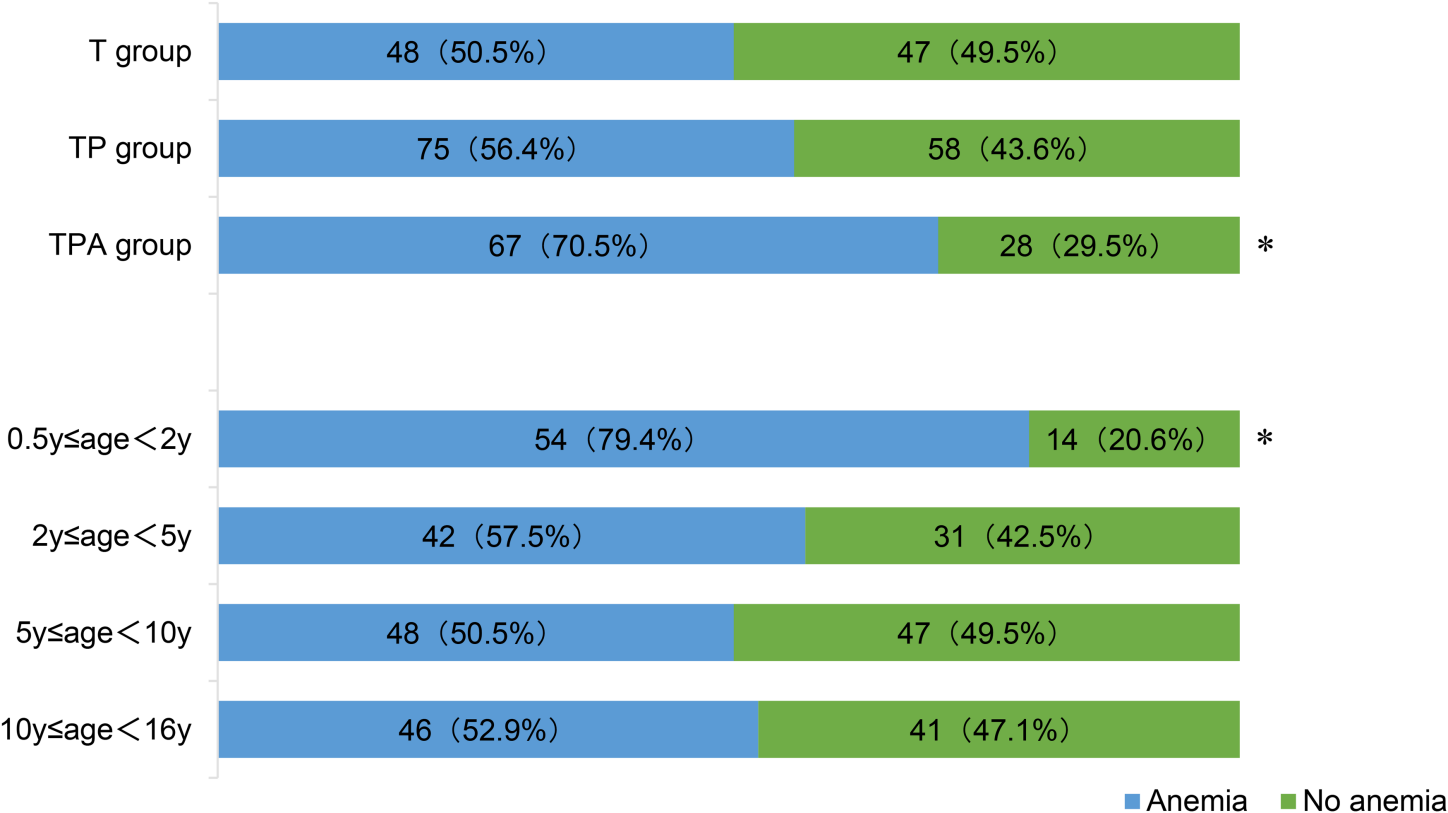
Anemia in children over 6 months of age. *Significant difference from other groups (p < 0.05). Anemia was defined as Hb concentration: (1) children aged 6–59 months: Hb<110 g/L; (2) children aged 5–11 years: Hb<115 g/L; and (3) children aged 12–16 years: Hb<120 g/L.

Figure 3 showed the incidence of anemia among children over 6 months of age. Children aged 0.5–2 years exhibited the highest anemia prevalence of 70.6% (48/68) among the four age groups (*P *< 0.05). The TPA group had the highest incidence of anemia (70.5%, 67/95) compared to T group and TP group (*P *< 0.05).

### 3.4. Basic descriptions, biochemical indexes and physical characteristics of children in the treatment group and abandonment group

As shown in Table 4, the basic characteristics of the children, including region, sex, diagnosis and guardians' education were not significantly different between the 2 groups (*P* > 0.05). However, compared with the treatment group, children in the abandonment group were younger and had higher nutritional risk scores (*P* < 0.05). Furthermore, the BAZs, HAZs and concentrations of Hb and ALB in the abandonment group were lower than those in the treatment group, and the differences were statistically significant (*P* < 0.05, Table 4). Compared with the treatment group, the rate of wasting (50%) and the prevalence of anemia (68.2%) were higher among children over 6 months of age in the abandonment group (*P *= 0.0002, 0.005, respectively) (data not shown). The prevalence of severe malnutrition in the abandonment group (15/55, 27.3%) was higher than that in the treatment group (24/313, 7.7%) (*P* = 0.0002). No significant differences were observed in the prevalence of malnutrition between the two groups (*P *> 0.05).

**Table 4:** Basic descriptions, biochemical indicators and physical characteristics of children in the treatment group and abandonment group.

	Treatment (*n* = 313)	Abandonment (*n* = 55)	*X^2^* or *T*	*P*
Basic descriptions				
Age (year)	5.58 (1.50, 9.92)	2.25 (0.58, 6.33)	9.76	0.0018
Nutritional risk score (p25, p75)	3 (2, 4)	3 (3, 5)	9.03	0.0027
Region, *N* (%)				
Rural	270 (86.3)	52 (94.6)	2.93	0.087
Urban	43 (13.7)	3 (5.4)		
Guardians’ education, *N* (%)				
Junior middle school or below	265 (84.7)	41 (74.5)		
Senior high school or above	48 (15.3)	14 (25.5)	3.42	0.064
Sex, *N* (%)				
Boy	177 (56.5)	31 (56.4)	0.0007	0.98
Girl	136 (43.5)	24 (43.6)		
Diagnosis, *N* (%)				
TBM	79 (25.2)	20 (36.4)	3.78	0.15
TBM + PTB	134 (42.8)	17 (30.9)		
TBM + PTB + ATB	100 (31.9)	18 (32.7)		
Biochemical indicators (g/L)				
Hb	109.15 ± 16.43	101.22 ± 18.47	8.99	0.0027
TP	66.11 ± 8.36	63.95 ± 7.98	2.82	0.093
ALB	39.65 ± 6.43	31.4 ± 7.32	5.36	0.024
Cr	28.88 ± 11.37	27.73 ± 8.35	5.98	0.074
BUN	3.36 ± 1.31	3.02 ± 1.37	5.45	0.06
ALP	141.12 ± 63.61	138.36 ± 62.67	0.31	0.575
Anemia, *N* (%)^#^	144 (51.6)	30 (68.2)	10.1	0.005
Physical measures				
BAZ	−1.14 ± 1.34	−1.58 ± 1.72	5.1	0.024
HAZ	−0.24 ± 0.88	−0.79 ± 1.01	5.3	0.026
Degree of malnutrition, *N* (%)				
Mild	99 (31.6)	12 (21.8)		
Moderate	57 (18.2)	10 (18.2)	19.5	0.0002
Severe	24 (7.7)	15 (27.3)		
Malnutrition, *N* (%)	114 (57.5)	37 (67.3)	1.8	0.17

Hb, hemoglobin; TP, total protein; ALB, albumin; Cr, creatinine; BUN,blood urea nitrogen; ALP, salkaline phosphatase.

Anemia was defined as Hb concentration:children aged 6–59 months: Hb < 110 g/L; children aged 5–11 years: Hb < 115 g/L; and children aged 12–16 years: Hb < 120 g/L.

BAZ, BMI-for-age *z* score; HAZ, height-for-age *z* score.

Malnutrition, BAZ < −1; mild malnutrition, −2 ≤ BAZ < −1; moderate malnutrition, −3 ≤ BAZ < −2; severe malnutrition, BAZ < −3.

^#^ 323 children over 6 months had data on anemia, including 279 cases in the treatment group and 44 cases in the abandonment group.

*Significant difference from the treatment group, with *P *< 0.05.

### 3.5. Multiple logistic regression analysis of factors influencing guardians' treatment decisions

According to the results of the univariate analysis, parameters with statistical significance were included in the multiple logistic regression analysis. The results showed (Table 5) that age, malnutrition, anemia, nutritional risk degree, and diagnosis were significantly associated with guardians' decision for continued treatment, with a *P *<* *0.05. In particular, children who were older (*OR =* 1.1), had a higher BAZ (O*R *=* *1.98), and had no anemia (OR = 1.02) were more likely to obtain treatment with their guardians' support than those who were younger, had a lower BAZ, and suffered from anemia (*P *< 0.05). Children who had nutritional risk (OR = 0.56) were less likely to obtain treatment with their guardians' support than children who did not have nutritional risk (*P *< 0.05). TBM patients with PTB and/or ATB (OR = 0.65) were less likely to obtain treatment with their guardians' support than children with TBM (*P *< 0.05).

**Table 5:** The relationship between significant predictor variables and guardians’ treatment decisions.

Parameter	*B*	Standard error	Wald chi-square	*P*	OR	95% CI
Age	0.09	0.04	6.0	0.014	1.1	(1.02, 1.19)
BAZ	0.68	0.32	4.55	0.032	1.98	(1.06, 3.69)
Anemia	0.02	0.01	5.02	0.025	1.02	(1.003, 1.04)
Nutritional risk degree	−0.58	0.33	4.66	0.03	0.56	(0.29, 1.07)
Diagnosis	−0.5	0.21	5.68	0.017	0.65	(0.25, 1.05)

## 4. Discussion

As is known, malnutrition and anemia are common among children with TB.It has been reported that poor nutritional status was associated with increased extrathoracic TB (20). The aim of this survey was to explore the effects of TB infection at different sites on anthropometric indicators, malnutrition and anemia incidence in children in Southwest China.In this survey,the BAZs, HAZs, and concentrations of Hb among children with TB were decreased, the prevalence of malnutrition and anemia was 58.7% (216/368)and 58.8% (190/323), respectively.These findings suggest that nutritional status is a serious public health problem among children with TBM in Southwest China. Furthermore, this survey was the first compared the nutritional status of children with TB infection at different sites.

Existing data demonstrate that malnutrition is common among children with TB (6, 7, 9, 21) and vice versa (22, 23), with mortality tending to increase when these two diseases co-occur (24). In our survey, children in the TPA group had a higher incidence of malnutrition, reaching 69.5% (82/118). This suggests that children were more likely to suffer from malnutrition when these three diseases (TBM, PTB and ATB) co-existed. Intestinal TB involving the ileocecal region and ulcerative type mainly presents with malabsorption syndrome.Poor nutrition could be caused by decreased intake, malabsorption and increased loss (due to vomiting, abdominal pain, diarrhea, etc.).

Infancy (children aged 1 month to 2 years) and adolescence (children aged 10–16 years) are the two growth and TB infection peaks (24–26). Therefore, it is likely that infants and adolescents are more susceptible to stunted growth when TB occurs. However, contrary to other reports (3, 13, 27, 28), the prevalence of malnutrition was not the highest in children under 2 years of age in our survey. We speculate that they may have benefited from national policies. In 2017, the General Office of the State Council of the People's Republic of China issued the National Nutrition Plan of Action (2017–2030) (29), which explicitly proposed attaching importance to the nutrition of children in the first 1,000 days of life (within 2 years after birth) and formulated a series of measures. The nutritional status of children aged 10–16 years was the worst, with the malnutrition rate reaching 72.4% (63/87), which were higher than children at other ages. Adolescence is the second growth peak. they have a rapid growth in height and weight, and need more nutrition than children of other ages.Compared with healthy children of the same age, children in the 16 year-olds received fewer nutrients but consume more due to disease factors.There was no difference in the incidence of malnutrition between children at 2–10 years and those under 2 years. Some research showed that children at 2–10 years old,especially younger school age children (5–10 years old) seem to be relatively protected against TB, prior to a second peak in incidence during adolescence (24, 30). TB infection between 5 and 10 years of age rarely progressed to serious disease, and such progression was associated with significant clinical symptoms.The interactions between human host and the mycobacterium tuberculosis are extremely complex and dynami, and the true correlates of protective paediatric immunity remain unknown.

Anemia is a major health concern especially in developing countries. Children and women of reproductive age are especially susceptible.The main causes of anemia are infection, nutrition (iron deficiency disorder), bleeding and so on.TB and anaemia are mutually reinforcing and increase the prevalence of each other (31, 32).Children with TB may have a higher incidence of anaemia than healthy children due to infection and reduced nutrient intake (33–35). Anemia is an important factor affecting the growth and health of children, as well as the prognosis of diseases. However, there are few reports about anemia in children with TBM—the most serious type of TB. Our results showed that the incidence of anemia in the TPA group and 0.5∼ to 2-year-old group was the highest, reaching 70.5% and 79.4%, respectively. TB can induce systemic inflammation, and anemia of inflammation may worsen with an increase in the number of infected sites. Compared with adolescents, infants and young children with TBM have an acute onset and rapid progression (36), which may prompt them to seek medical treatment earlier. Malnutrition often takes a certain amount of time to occur, while anemia may occur at any time.

In this survey,it was showed that some TB patients eventually abandoned treatment. Some researchers reported that low income and education were the reasons of abandoning treatment (37). Unfortunately, most of the guardians in this survey were unwilling to provide annual household income due to privacy issues, it was collected the regional distribution (including rural and urban) to reflect the economics in this survey. There was no statistical difference in regional distribution and education level between the abandonment group and the treatment group (*P* > 0.05, Table 4). This results were inconsistent with others’ (37), which might be attributed to the fact that the patients’ guardians were mainly from poor areas in Southwest China, who were at a low socioeconomic level and low education level. Furthermore, it had not been reported that whether nutritional status was a factor that affected guardians'decision to treat (abandonment or treatment).This survey was the first reported the nutritional status of children in the abandonment group and the treatment group. It showed that nutritional status was one of the causes of abandoning treatment in children with TB infection in Southwest China.

## 5. Conclusions

This cross-sectional survey showed that the nutritional status of children with TB at different sites in Southwest China was seriously affected. Malnutrition and anemia were common among TBM patients, especially those combined with PTB and ATB. The prevalence rates of anemia and malnutrition were the highest among children aged 0.5–2 years and 10–16 years, respectively. Therefore, in clinical practice, we should focus on monitoring anthropometric indicators and biochemical indicators to detect growth deviation and anemia as early as possible, and carry out standardized nutrition management.

## Data Availability

The original contributions presented in the study are included in the article, further inquiries can be directed to the corresponding author.
