# Supplementary material for: Growth and anemia among children with tuberculosis infection at different sites in Southwest China
Source: Front Pediatr. 2023 Jun 15;11:1188704. doi: 10.3389/fped.2023.1188704 (PMC10309554; doi:10.3389/fped.2023.1188704)
Supplement: Supplementary file 1 [file Table1.docx]

Supplementary Material

Growth and anemia among children with tuberculosis infection at different sites in Southwest China

Zhongmin Gao^1^,Quanbo Liu^2^ , Qin Deng^1^,Lin Kong^1^ , Yongfang Liu^1*^

*** Correspondence:**Yongfang Liu:liuyongfang811@163.com

# Supplementary Figures and Tables

## Supplementary Figures


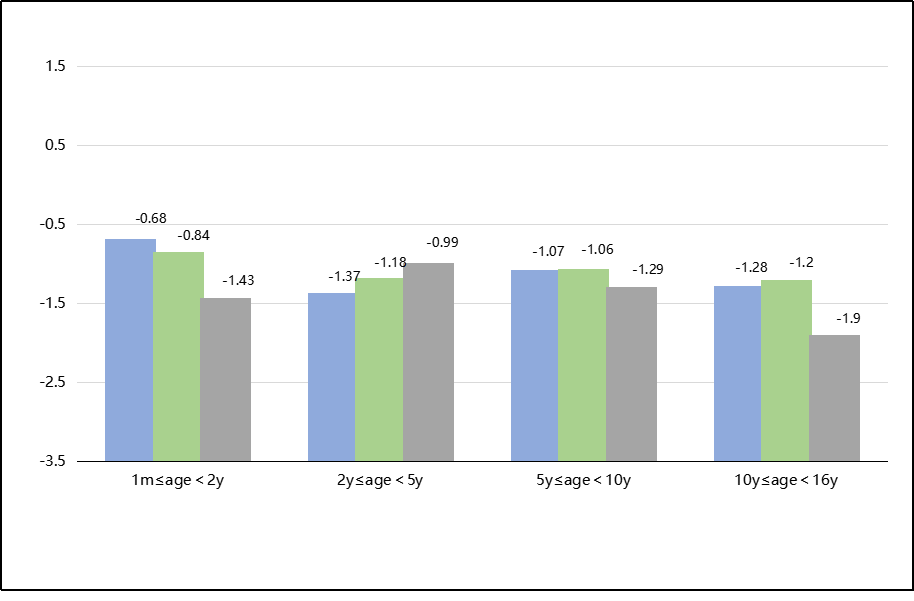


*

**BAZ**

^†^

^†^

T group

TPA group

TP group

FIGURE 1 | Comparison of BAZ levels in three groups at different ages. BAZ,body mass index(BMI)-for-age z score.*Significant difference from other groups (*p* < 0.05).


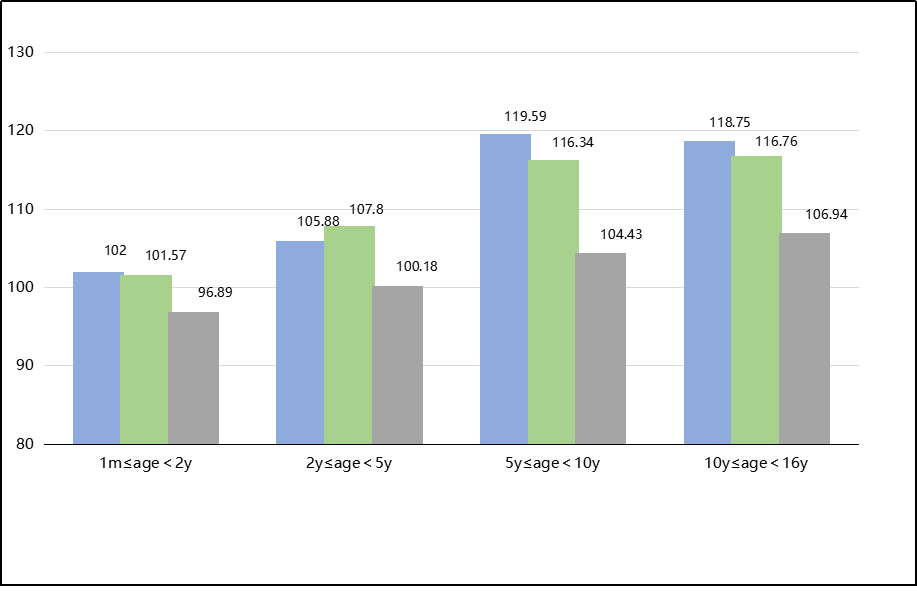


*

*

**HB(g/L)**

^†^

^†^

^†^

^†^

T group

TPA group

TP group

FIGURE 2 | Comparison of HB levels in three groups at different ages. Hb,hemoglobin

*Significant difference from other groups (*p* < 0.05).


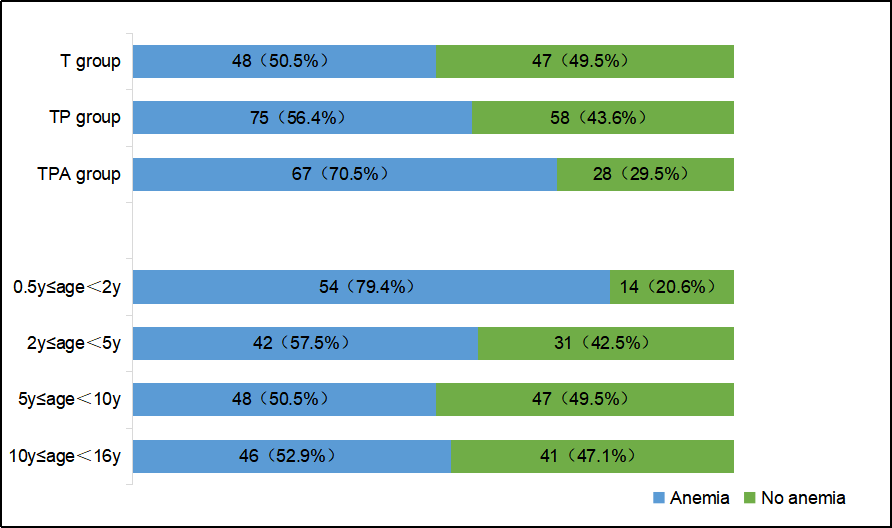


*

*

FIGURE 3 |Anemia in children over 6 months of age.

*Significant difference from other groups (*p* < 0.05).

Anemia was defined as Hb concentration: (1) children aged 6-59 months: Hb<110 g/L; (2) children aged 5-11 years: Hb<115 g/L; and (3) children aged 12-16 years: Hb<120 g/L.

## Supplementary Tables

Table 1 BAZ, HAZ and biochemical indicators among patients in different groups.

| Parameter | T group ^*^  (n=99) | TP group ^†^  (n=151) | TPA group ^#^  (n=118) | *P*^*^ ^†^ | *P* ^*^ ^#^ | *P*^†^ ^#^ |
| --- | --- | --- | --- | --- | --- | --- |
| Age（y） | 5.75（3.08-9.33） | 4.08（0.91-9.50） | 4.79（0.80-11.42） | 0.23 | 0.68 | 0.42 |
| BAZ | -1.13±1.36 | -1.14±1.48 | -1.47±1.31 | 0.63 | 0.04 | 0.01 |
| HAZ | -0.03±0.77 | -0.25±0.87 | -0.52±0.98 | 0.05 | <0.001 | 0.02 |
| Hb（g/L) | 112.98±15.10 | 109.55±15.33 | 101.73±18.61 | 0.11 | <0.001 | 0.0001 |
| TP（g/L) | 65.09±6.48 | 66.84±7.82 | 65.02±10.09 | 0.10 | 0.95 | 0.07 |
| ALB（g/L) | 41.63±4.97 | 40.43±5.60 | 36.40±7.76 | 0.13 | <0.001 | <0.001 |
| Cr（g/L) | 26.47±9.93 | 28.49±11.87 | 29.47±10.77 | 0.16 | 0.05 | 0.47 |
| BUN（g/L) | 3.14±1.30 | 3.43±1.33 | 3.23±1.35 | 0.09 | 0.62 | 0.22 |
| ALP（g/L) | 134.87±55.54 | 142.31±61.07 | 143.54±72.12 | 0.36 | 0.32 | 0.87 |

BAZ, body mass index-for-age z score;HAZ,height-for-age z score;Hb, hemoglobin; TP,total protein ; ALB, albumin ; Cr,creatinine;BUN,blood urea nitrogen ;ALP, salkaline phosphatase

Table 2 HAZ and biochemical indicators among children of different ages.

|  | 1 m≤age<2 y |  |  | 2 y≤age<5 y |  |  | 5 y≤age<10 y |  |  | 10 y≤age<16 y |  |  |
| --- | --- | --- | --- | --- | --- | --- | --- | --- | --- | --- | --- | --- |
| Parameters | T group  (n=16) | TP group  (n=53) | TPA group  (n=44) | T group  (n=26) | TP group  (n=30) | TPA group  (n=17) | T group  (n=37) | TP group  (n=35) | TPA group  (n=23) | T group  (n=20) | TP group  (n=33) | TPA group  (n=34) |
| HAZ | -0.40±0.58 ^*^ | -0.58±1.03 ^*^ | -1.04±1.22^†^ | -0.11±0.78 | 0.09±0.74 | -0.26±0.87 | 0.21±0.77 | 0.15±0.62 | -0.09±0.66 | -0.06±0.77 ^*^ | -0.14±0.38 ^*^ | -0.97±0.47^†^ |
| TP（g/L) | 62.7±6.47 | 65.54±7.28 | 64.47±9.17 | 63.02±6.36 | 67.56±7.43 | 64±7.6 | 65.87±4.98 | 66.54±7.87 | 63.11±10.67 | 68.23±7.81 | 68.6±8.83 | 67.54±11.71 |
| ALB （g/L) | 39.26±5.46 | 39.46±5.5 | 36.63±7.28 | 40.06±5.15 | 40.91±5.47 | 38.26±8.18 | 43±3.88 | 41.06±4.67 | 35.48±8.86 | 43.06±5.14 ^*^ | 40.89±6.74 ^*^ | 35.78±7.54^†^ |
| Cr（g/L) | 17.64±6.41 | 19.75±4.57 | 22.59±7.5 | 22.57±5.43 | 24.24±5.86 | 25.65±5.07 | 29.07±8.53 | 30.45±7.48 | 31.59±8.86 | 33.79±11.96 | 44.32±11.42 | 38.87±10.6 |
| BUN（g/L) | 2.31±1.32 | 3.05±1.24 | 3.04±1.44 | 2.94±1.26 | 3.26±1.35 | 3.21±1.28 | 3.51±1.29 | 3.75±1.43 | 3.36±1.26 | 3.39±1.18 | 3.85±1.17 | 3.4±1.34 |
| ALP （g/L) | 161.75±65.91 | 169.61±68.73 | 191.03±86.38 | 120.05±34.72 | 131.15±41.75 | 133.87±48.21 | 127.71±35.75 | 129.94±55.73 | 127.8±42.62 | 145.88±85.66 | 121.74±54.54 | 97.58±31.19 |

^*,†^Values within a row with different superscript characters are significantly different among the corresponding groups, with *P*<0.05.HAZ,height-for-age z scoreTP,total protein ;ALB, albumin ; Cr,creatinine;BUN,blood urea nitrogen ;ALP, salkaline phosphatase

Table 3 Malnutrition among the patients.

|  | Group | | | Age | | | | **P* | ^#^*P* |
| --- | --- | --- | --- | --- | --- | --- | --- | --- | --- |
|  | T group  (n=99) | TP group  (n=151) | TPA group  (n=118) | 1 m≤age<2 y  (n=113) | 2 y≤age<5 y  (n=73) | 5 y≤age<10y  (n=95) | 10 y≤age<16 y  (n=87) |  |  |
| Degree of malnutrition |  |  |  |  |  |  |  |  |  |
| Mild n(%) | 30（30.3） | 39(25.8) | 42(35.6) | 33（29.2） | 19（26） | 28（29.5） | 31（35.6） | 0.15 | 0.17 |
| Moderate n(%) | 16（16.2） | 27(17.9) | 24(20.3) | 14（12.4） | 10（13.7） | 17（17.9） | 26（29.9） |  |  |
| Severe n(%) | 8（8.1） | 14(9.3) | 16(13.6) | 13（11.5） | 11（15.1） | 9（9.5） | 6（6.9） |  |  |
| Malnutrition n(%) | 54（54.5）^*^ | 80（53）^*^ | 82（69.5）^†^ | 60(53.1)^*^ | 40(54.8)^*^ | 54(56.8)^*^ | 63(72.4)^†^ | 0.001 | 0.03 |

^*,†^Values within a row with different superscript characters are significantly different among the corresponding groups, with *P*<0.05.

**P*: Comparison of the T group, TP group, and TPA group; #*P*: Comparison of the four age groups.

Malnutrition,BAZ<-1;mild malnutrition,-2≤BAZ<-1; moderate malnutrition,-3≤BAZ<-2 ; severe malnutrition, BAZ<-3

Table 4 Basic descriptions, biochemical indicators and physical characteristics of children in the treatment group and abandonment group

|  | Treatment (n=313) | Abandonment (n=55) | *X^2^* or *T* | *P* |
| --- | --- | --- | --- | --- |
| **Basic descriptions** |  |  |  |  |
| Age（y） | 5.58（1.50,9.92） | 2.25（0.58,6.33） | 9.76 | 0.0018 |
| Nutritional risk score (p25, p75) | 3（2,4） | 3（3,5） | 9.03 | 0.0027 |
| Region N(%) |  |  |  |  |
| Rural | 270（86.3） | 52（94.6） | 2.93 | 0.087 |
| Urban | 43（13.7） | 3（5.4） |  |  |
| guardians' education N(%) |  |  |  |  |
| Junior middle  school or below | 265(84.7) | 41(74.5) | 3.42 | 0.064 |
| Senior high school or above | 48(15.3) | 14(25.5) |  |  |
| Sex N(%) |  |  |  |  |
| Boy | 177（56.5） | 31（56.4） | 0.0007 | 0.98 |
| Girl | 136（43.5） | 24（43.6） |  |  |
| Diagnosis N(%) |  |  |  |  |
| TBM | 79（25.2） | 20（36.4） | 3.78 | 0.15 |
| TBM+PTB | 134（42.8） | 17（30.9） |  |  |
| TBM+PTB+ATB | 100（31.9） | 18（32.7） |  |  |
| **Biochemical indicators** (g/L) |  |  |  |  |
| Hb | 109.15±16.43 | 101.22±18.47 | 8.99 | 0.0027 |
| TP | 66.11±8.36 | 63.95±7.98 | 2.82 | 0.093 |
| ALB | 39.65±6.43 | 31.4±7.32 | 5.36 | 0.024 |
| Cr | 28.88±11.37 | 27.73±8.35 | 5.98 | 0.074 |
| BUN | 3.36±1.31 | 3.02±1.37 | 5.45 | 0.06 |
| ALP | 141.12±63.61 | 138.36±62.67 | 0.31 | 0.575 |
| Anemia N(%)^#^ | 144(51.6) | 30(68.2) | 10.1 | 0.005 |
| **Physical measures** |  |  |  |  |
| BAZ | -1.14±1.34 | -1.58±1.72 | 5.1 | 0.024 |
| HAZ | -0.24±0.88 | -0.79±1.01 | 5.3 | 0.026 |
| Degree of malnutrition N(%) |  |  |  |  |
| Mild | 99（31.6） | 12（21.8） | 19.5 | 0.0002 |
| Moderate | 57（18.2） | 10（18.2） |  |  |
| Severe | 24（7.7） | 15（27.3）* |  |  |
| Malnutrition N(%) | 114（57.5） | 37（67.3） | 1.8 | 0.17 |

^#^ 323 children over 6 months had data on anemia, including 279 cases in the treatment group and 44 cases in the abandonment group. *Significant difference from the treatment group, with *P*<0.05.

Hb, hemoglobin; TP,total protein ;ALB, albumin ; Cr,creatinine;BUN,blood urea nitrogen ;ALP, salkaline phosphatase.

Anemia was defined as Hb concentration:children aged 6-59 months: Hb<110 g/L; children aged 5-11 years: Hb<115 g/L; and children aged 12-16 years: Hb<120 g/L.

BAZ, BMI-for-age z score;HAZ,height-for-age z score.

Malnutrition,BAZ<-1;mild malnutrition,-2≤BAZ<-1; moderate malnutrition,-3≤BAZ<-2 ; severe malnutrition, BAZ<-3.

Table 5 The relationship between significant predictor variables and guardians’ treatment decisions.

| **Parameter** | ***B*** | **Standard error** | **Wald chi-square** | **P** | **OR** | **95% CI** |
| --- | --- | --- | --- | --- | --- | --- |
| Age | 0.09 | 0.04 | 6.0 | 0.014 | 1.1 | (1.02,1.19) |
| BAZ | 0.68 | 0.32 | 4.55 | 0.032 | 1.98 | (1.06,3.69) |
| Anemia | 0.02 | 0.01 | 5.02 | 0.025 | 1.02 | (1.003,1.04) |
| Nutritional risk degree | -0.58 | 0.33 | 4.66 | 0.03 | 0.56 | (0.29,1.07) |
| Diagnosis | -0.5 | 0.21 | 5.68 | 0.017 | 0.65 | (0.25,1.05) |

BAZ, body mass index-for-age z score;Anemia was defined as Hb concentration:children aged 6-59 months: Hb<110 g/L; children aged 5-11 years: Hb<115 g/L; and children aged 12-16 years: Hb<120 g/L.
